# Association Between Vitamins and Amyotrophic Lateral Sclerosis: A Center-Based Survey in Mainland China

**DOI:** 10.3389/fneur.2020.00488

**Published:** 2020-06-18

**Authors:** Mengli Wang, Zhen Liu, Weining Sun, Yanchun Yuan, Bin Jiao, Xuewei Zhang, Lu Shen, Hong Jiang, Kun Xia, Beisha Tang, Junling Wang

**Affiliations:** ^1^Department of Neurology, Xiangya Hospital, Central South University, Changsha, China; ^2^General Practice, The Fifth Affiliated Hospital, Sun Yat-sen University, Zhuhai, China; ^3^Health Management Center, Xiangya Hospital, Central South University, Changsha, China; ^4^Laboratory of Medical Genetics, Central South University, Changsha, China; ^5^Key Laboratory of Hunan Province in Neurodegenerative Disorders, Central South University, Changsha, China; ^6^National Clinical Research Center for Geriatric Diseases, Xiangya Hospital, Central South University, Changsha, China

**Keywords:** vitamin, amyotrophic lateral sclerosis, neurodegenerative disease, risk factors, correlation

## Abstract

To date, conflicting results about the role of vitamins in amyotrophic lateral sclerosis (ALS) have been reported along with a lack of systematic studies on all types of serum vitamins in patients with ALS. Moreover, extensive studies have been conducted on vitamins in other neurodegenerative diseases; however, whether serum vitamin alterations in ALS are similar to those in other neurodegenerative diseases remains unclear. Therefore, we performed a study involving a large Chinese cohort of patients with ALS to address this gap. In this study, 202 patients with ALS, 214 with a neurodegenerative disease that mimicked ALS (mimics), and 208 healthy controls were enrolled. Serum vitamins of all subjects were examined under fasting state in Clinical Laboratory. As a result, we found that higher vitamin A and E levels and lower vitamin B2, B9, and C levels were in patients with ALS compared to healthy controls, and that high vitamin A and E levels, and low vitamin B2, B9, and C levels were associated with an increased risk for ALS. In addition, serum vitamin C was lower in early-onset ALS patients compared to those in late-onset ALS patients; however, there was no significant correlation between serum vitamins and age at onset, sites at onset, disease duration, or disease severity of ALS. We also found that patients with ALS showed similar vitamin alterations to mimics, with the exception of vitamin E. In summary, our study adds information to the literature on the role of vitamins in ALS and provides support for clinical guidance regarding dietary changes and vitamin supplements in patients with ALS.

## Introduction

Amyotrophic lateral sclerosis (ALS) is an intractable neurodegenerative disease characterized by progressive degeneration of the upper and lower motor neurons at the motor cortex, bulbar, and spinal levels. The typical symptoms of ALS include progressive weakness, muscle atrophy, cramps, fasciculation, and spasticity (1). Patients usually die from respiratory failure within 3–5 years after disease onset. The worldwide prevalence of ALS is 4.42 per 100,000 population and increases with age until the age of 70–79 years (2), with established risk factors being age, male sex, and family history (3). About 10% of ALS is familial, leaving 90% of patients with the sporadic form of ALS. Whilst the etiology and pathology of ALS remain unclear, current evidence suggests that oxidative stress, glutamatergic toxicity, dysfunction of ribonucleic acid (RNA) processing, and abnormal protein aggregation might play important roles in the pathogenesis of ALS (4).

Unfortunately, there is no therapy available that will cure ALS (5). Limited understanding of ALS pathophysiology is a major obstacle to the development of effective therapies. Knowledge about risks and prognostic factors for ALS may help uncover molecular pathways that could serve as novel therapeutic targets (6, 7). Vitamins (important micronutrients) play a critical role in the development of the nervous system. The possibility of different vitamins to serve as risks, prognostic factors, or in the pathogenesis of ALS has been extensively researched (6, 8–18). Several large population studies demonstrated that regular vitamin E supplementation, not vitamin C, β-carotene, or multivitamins, was associated with a lower risk of ALS (17, 19, 20). A pooled analysis from five prospective studies further support that long-term vitamin E supplementation was associated with lower ALS prevalence (18). However, Okamoto et al. (21) observed no statistically significant dose-response relationship between vitamin E, ascorbic acid, and β-carotene intake and the risk of ALS. In addition, de Bustos et al. (22) found that there was no difference in cerebrospinal fluid (CSF) and serum vitamin E levels between ALS patients and matched controls. Results pooled from five cohort studies revealed that carotenoid intake, not vitamin C, was associated with a reduced risk of ALS (11). However, treatment with retinoids resulted in a negative effect on the survival of an animal ALS model (10). Moreover, there was no difference in vitamin A levels between ALS patients and normal controls in the study by Molina et al. (23). Several studies suggested that vitamin D supplementation has a beneficial effect on the prognosis of ALS. However, the neuroprotective and survival benefits of vitamin D for ALS remain elusive, as the results of some studies are controversial (8, 9, 24–26). Zhang et al. (27) found that folic acid levels were significantly decreased at the middle to the late stages of the disease in SOD1^G93A^ transgenic mice. They also revealed that combined treatment with folic acid and vitamin B12 could significantly delay the disease onset and prolong the lifespan of an ALS mouse model by attenuating plasma homocysteine levels, suppressing microglial and astrocytes activation, and inhibiting inducible nitric oxide synthase and tumor necrosis factor-alpha expression. Intraperitoneal ultra-high dosage of methylcobalamin was found to inhibit the disease progression in an ALS mouse model (12). Moreover, a recent long-term phase II/III randomized controlled study concluded that ultra-high doses of methylcobalamin might retard symptomatic progression and prolong the survival of ALS patients if started early. However, there was no significant efficacy in the whole cohort (14). Generally speaking, though some studies have shown an association between vitamins and ALS, some results are controversial. Additionally, studies have reported that the majority of water-soluble vitamins (such as vitamin B1, B2, B9, B12, and C), in their biologically active forms, work synergistically as essential coenzymes in several biochemical pathways in the brain that are essential to the development, myelination, and proper function of the central and peripheral nervous systems (28–30). Therefore, it is necessary to take into account all vitamins at the same time when exploring serum vitamins. However, there has been a lack of systematic studies that evaluate all types of serum vitamins in the same cohort of ALS patients.

ALS and other neurodegenerative diseases share similar pathogenic pathways, such as excitotoxicity, oxidative stress, and abnormal protein aggregates. The effect of vitamins on other neurodegenerative diseases also has been extensively investigated. Studies in animal models of Alzheimer's disease (AD) revealed that ascorbic acid could restore behavioral deficits, memory impairment, and the reduction in brain synaptophysin, as well as reduce the formation of amyloid-β oligomers, phosphorylation of Tau at Ser39, oxidative stress markers, and proinflammatory cytokines (31–34). Clinical studies found that plasma ascorbate levels were lower in AD patients (35) and that the combined supplementation with ascorbic acid and vitamin E, not ascorbic acid alone, was associated with a reduced prevalence and incidence of AD (36–40). Although different leukocyte ascorbic acid levels were found between patients with Parkinson's disease (PD) and age-matched controls (41), similar serum ascorbic acid levels were reported in patients with PD and healthy individuals (42). In addition, several prospective studies suggested that intake of vitamin C, vitamin E, and carotenoids did not substantially affect the risk of PD (43–45). Low vitamin D serum levels were found in patients affected by PD, AD, and multiple sclerosis (MS). The association between low vitamin D serum levels and the risk of AD and PD has also been reported (46–52). However, a contradictory finding that vitamin D supplementation has no impact in AD and PD also exists (53–55). Although a recent meta-analysis demonstrated the beneficial effects of vitamin E on preventing AD (56), a double-blind, randomized clinical trial conducted for 6 years on a population of 7,540 men who were at least 60 years old found that supplementation with vitamin E did not prevent AD (57). Several studies reported low serum and plasma levels of vitamin A, α-carotene, β-carotene, lycopene, and lutein in patients with AD (58–61), whereas other studies demonstrated conflicting results about plasma α-carotene, β-carotene, lycopene, and lutein levels in patients with AD (62–64). Lower plasma vitamin A levels have also been reported in patients with PD (65). In addition, serum levels of α- and β-carotenes were inversely correlated with the Hoehn and Yahr stage and United Parkinson's Disease Rate Scale (UPDRS) motor score in PD patients (66). A meta-analysis of ten studies revealed that PD patients had lower vitamin B12 levels than controls, and higher dietary intake of vitamin B6 was associated with a decreased risk of PD, while no significant association was observed for dietary intake of vitamin B12 and folate and the risk of PD (67).

Generally speaking, though vitamins are suggested to play important roles in ALS, controversial results make it necessary to research them further. There have been extensive studies of vitamins in other neurodegenerative disease, but whether the trend of serum vitamin alterations in ALS is similar to that in other neurodegenerative diseases remains unclear. In addition, some vitamins work synergistically; however, there is a lack of systematic studies that research all types of serum vitamins at the same time in patients with ALS and with other neurodegenerative diseases. For the above reasons, this study has the following aims: (1) to compare serum levels of different vitamins (vitamin A, B1, B2, B9, B12, C, D, and E) among patients with ALS, patients with other neurodegenerative diseases (mimics), and healthy controls; (2) to explore the relationship between vitamins and the risk for ALS; (3) to explore the associations between vitamin levels and clinical characteristics of ALS, including age at onset (AAO), sites of onset, disease duration, and ALS functional rating scale revised (ALSFRS-R).

## Materials and Methods

### Subjects

A total of 202 consecutive patients with ALS of Chinese Han ancestry were recruited from April 30, 2013, to April 30, 2019. All patients with ALS were diagnosed with definite, probable, or probable-laboratory-supported ALS by at least two neurologists according to the revised El Escorial criteria 2015 (68). During the same period, 214 gender- and age-matched patients with a diagnosis of another neurodegenerative disease [including 116 patients with PD, 15 patients with AD, 75 patients with multiple system atrophy (MSA), and eight patients with spinocerebellar ataxia (SCA)], and 208 matched healthy people were enrolled as mimics and healthy controls, respectively. Patients with ALS and mimics were from the Department of Neurology, Xiangya Hospital, Central South University (CSU), China, and healthy controls were from the Health Management Center, Xiangya Hospital, CSU, China. All patients with PD were diagnosed according to the MDS Clinical Diagnostic Criteria (69). All patients with AD met the diagnostic criteria of clinical probable AD established by the National Institute of Neurological and Communicative Disorders and Stroke and the Alzheimer's Disease and Related Disorders Association (NINCDS-ADRDA) in 2007 (70). All patients with MSA were diagnosed according to the current consensus criteria established by Gilman and colleagues (71). All patients with SCA met the criteria of either clinical or genetic diagnosis of SCA. Healthy controls had no neurological diseases. The exclusion criteria included (1) people with digestive system disease, renal failure, anemia, peripheral neuropathy, disabling stroke, clinical depression, malignant tumor, and chronic wasting disease; (2) people who were receiving vitamin supplementation; (3) people who had ALS and other neurodegenerative diseases at the same time; (4) people who were overweight or severely malnourished. In addition, all subjects enrolled in this study were from Hunan Province and shared similar dietary habits. The study was approved by the Expert Committee of Xiangya Hospital, Central South University in China and all included subjects provided written informed consent prior to participation.

The demographic and clinical data, including name, gender, age, education level, smoking, drinking, family history, site of onset, AAO, disease duration, and ALSFRS-R, were collected by specialists. If the patient had been hospitalized many times, the first hospitalization record was taken for registration.

### Biochemical Methods

Ten milliliters of peripheral venous blood samples were collected from all subjects after they had fasted for 8–12 h. Then, serum vitamins (including vitamin A, B1, B2, B9, B12, C, D, and E) and serum cholesterol were examined in the Clinical Laboratory, Xiangya hospital.

### Statistical Analysis

All analyses were performed using SPSS 22.0 (SPSS, Chicago, IL, USA) and GraphPad Prism 5.03 (®GraphPad Software, Inc.). Differences with *p* < 0.05 were considered to be statistically significant. Multiple comparisons were corrected with Bonferroni correction. A Chi-square test was used to analyze the difference in gender and the occurrence of menopause among different groups. The differences in vitamin levels, serum cholesterol, disease duration, and age between two different groups were analyzed with a Student's *t*-test. If more than two groups were compared, a one-way analysis of variance was used, followed by Dunn's multiple comparison test. The odds ratios (ORs) and 95% confidence intervals (CIs) of each serum vitamin were assessed using a binary logistic regression model. In the logistic regression, the dependent variable was a binary variable for which healthy control was coded as 0, and patient with ALS was coded as 1; independent variables consisted of each vitamin researched in the present study; control variables included age, gender, and serum cholesterol. All variables were treated as continuous except for gender and the presence or absence of ALS, which were binary. To check the multicollinearity in the model, the interaction between each variable was analyzed with Spearman's correlation analyses. Additionally, the variance inflation factors (VIF) of each variable were analyzed. The goodness fit of this model was evaluated by Prob > Chi2, Pseudo R2, and the Hosmer and Lemeshow test. The correlations between AAO, disease duration, ALSFRS-R score, and vitamin levels in patients with ALS were analyzed using Pearson's or Spearman's correlation analyses where appropriate.

## Results

### Demographic and Clinical Features of Subjects

The demographic and clinical features of subjects are listed in Table 1. Values are shown as means (±standard deviations) or percentages. The AAO of patients with ALS ranged from 13 to 81 years, with an average of 54.32 ± 10.36 years. The disease duration of ALS ranged from 1 to 120 months, with an average of 16.80 ± 17.23 months. The ALSFRS-R score ranged from 24 to 46, with an average of 39.96 ± 4.49. As for site at onset, 33.2 and 36.0% of patients with ALS had upper extremity and lower extremity onset, respectively, and 87.2% of patients had spinal cord symptoms at the time of onset. Among the 202 patients with ALS, three patients had a family history of ALS, accounting for 1.5% of the total patient number.

**Table 1:** Characteristics of patients with amyotrophic lateral sclerosis (ALS), mimics, and healthy controls.

	**Patients with ALS** **(*n* = 202)**	**Mimics** **(*n* = 214)**	**Healthy controls** **(*n* = 208)**	***p*-value**
Age (years)	55.68 ± 10.77	56.20 ± 12.32	55.71 ± 10.59	0.873
Gender, *n* (%)				0.637
Male	136 (67.3%)	137 (59.3%)	139 (66.8%)	
Female	66 (32.7%)	77 (40.7%)	69 (33.2%)	
Onset age (years)	54.32 ± 10.36	51.45 ± 13.63	–	
Disease duration (months)	16.80 ± 17.23	47.94 ± 51.52	–	
ALSFRS-R score	39.96 ± 4.49	–	–	
**Onset site**, ***n*** **(%)**				
Spinal	176 (87.1%)	–	–	
Bulbar	26 (12.9%)	–	–	
**EI Escorial category**, ***n*** **(%)**				
Possible	54 (26.7%)	–	–	
Probable	90 (44.6%)	–	–	
Definite	58 (28.7%)	–	–	
Education time (years)	8.78 ± 3.98	8.88 ± 3.78	–	
**Education**, ***n*** **(%)**				
Low (<9 years)	137 (67.8%)	104 (69.8%)	–	
High (>9 years)	65 (32.2%)	45 (30.2)	–	
**Smoking**, ***n*** **(%)**				
Never	125 (62.3%)	99 (66.4%)	–	
Ever or current	77 (37.7%)	50 (33.6%)	–	
**Drinking**, ***n*** **(%)**				
Never	126 (62.3%)	108 (72.4%)		
Ever or current	76 (37.7%)	41 (27.6%)		

*Values are shown as mean (±standard deviation) or percentages*.

### Vitamin Levels in Patients With ALS, Mimics, and Healthy Controls

**Figure 1:**
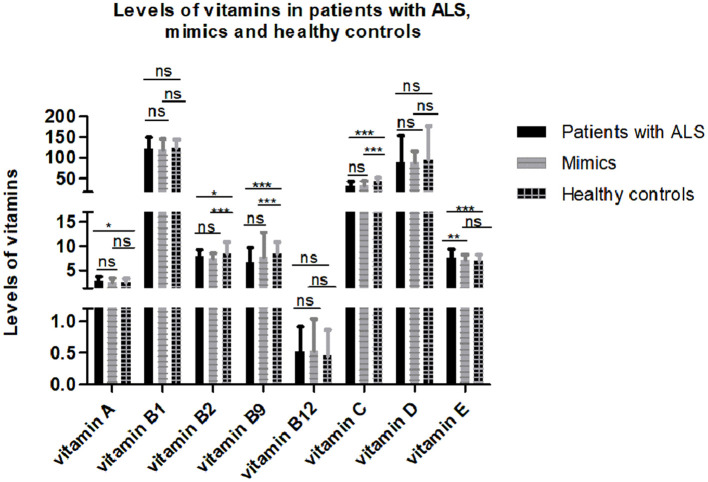
Levels of different vitamins in patients with amyotrophic lateral sclerosis (ALS), mimics, and healthy controls. Multiple comparisons were corrected with Bonferroni correction: ns denotes non-significant; * denotes *p* < 0.017; ** denotes *p* < 0.003; *** denotes *p* < 0.0003.

After controlling for age, gender, menopause, and serum cholesterol, serum vitamins were compared among 193 patients with ALS, 149 mimics, and 79 healthy controls. Some subjects without serum cholesterol data were excluded from the analysis. The results showed that serum levels of certain vitamins (vitamin A, B2, B9, C, and E) were significantly different among patients with ALS, mimics, and healthy controls (Figure 1 and Supplementary Table 1). Multiple comparisons revealed that higher levels of vitamin A and E and lower levels of vitamin B2, B9, and C were found in patients with ALS compared to those in healthy controls. There were no significant differences in vitamin A, B2, B9, and C levels between patients with ALS and mimics.

### Association Between Serum Vitamin Levels and the Risk of ALS

Adjusting for gender, age, and serum cholesterol, the association between serum vitamin levels and the risk of ALS were evaluated with a logistic regression model. The correlation coefficients between each variable were <0.25, indicating that there was no serious multicollinearity in the model (Supplementary Table 2). In addition, the VIF values of each variable were <2, further suggesting no serious multicollinearity in the model. The Prob > Chi2 was <0.0001, the value of Pseudo R2 was 0.328, and the *p*-value for Hosmer and Lemeshow test was 0.134, indicating that this model retains an acceptable fit to data. As shown in Table 2, higher serum vitamin B2, B9, and C were significantly associated with a reduced risk of ALS with the ORs (95%CI) being 0.78 (0.66–0.93), 0.85 (0.79–0.91), and 0.91 (0.89–0.95), respectively. However, higher serum vitamin A and E was significantly associated with an increased risk of ALS with the ORs (95%CI) being 1.66 (1.12–2.45) and 1.29 (1.02–1.61), respectively.

**Table 2:** Odd ratios (OR) of different vitamins for amyotrophic lateral sclerosis (ALS) adjusting for age, gender, serum cholesterol.

	**OR**	**95% CI**	***p*-value**
Vitamin A	1.66	1.12–2.45	**0.011**
Vitamin B1	1.01	0.99–1.02	0.106
Vitamin B2	0.78	0.66–0.93	**0.005**
Vitamin B9	0.85	0.79–0.91	**<0.001**
Vitamin B12	1.53	0.64–3.64	0.339
Vitamin C	0.92	0.89–0.95	**<0.001**
Vitamin D	1.00	0.99–1.00	0.158
Vitamin E	1.29	1.02–1.61	**0.028**

### Vitamins Levels and ALS Clinical Characteristics

**Figure 2:**
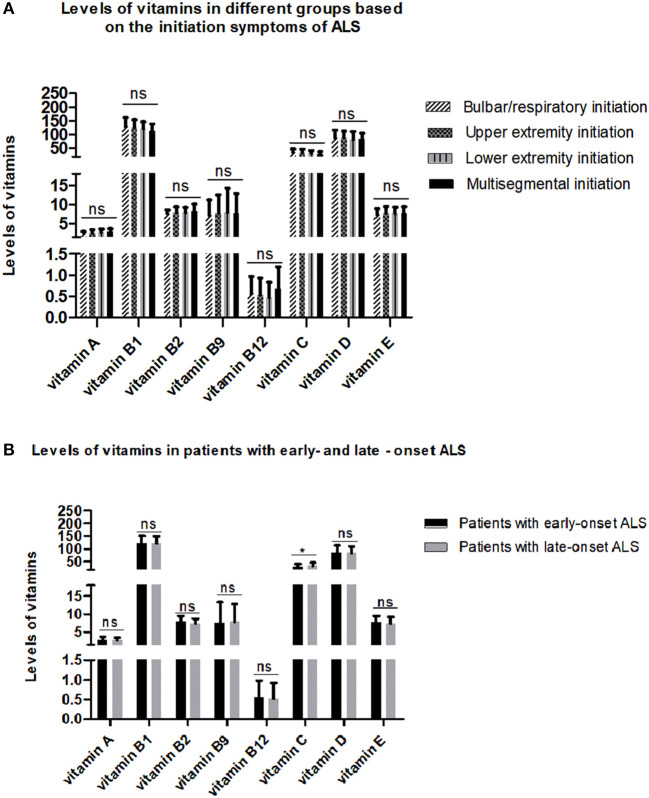
Levels of different vitamins in different amyotrophic lateral sclerosis (ALS) patient groups. **(A)** Patients with ALS were grouped by sites of disease onset. **(B)** Patients with ALS were divided by age at onset. ns denotes non-significant; * denotes *p* < 0.05.

To explore the relationship between serum vitamins and onset sites, we divided patients with ALS into four groups according to site of disease onset (bulbar, upper limb, lower limb, or multiple sites) and compared the levels of vitamins amongst the four groups after adjusting for gender, age, and disease duration; however, no significant differences were found (Figure 2A and Supplementary Table 3). We also compared the levels of vitamins between patients with early- and late-onset ALS after controlling for gender and disease duration, with the groups established according to whether the AAO of ALS was before or after 55 years of age. As shown in Figure 2B and Supplementary Table 4, lower levels of vitamin C were found in patients with early-onset ALS compared to those with late-onset ALS. We further analyzed vitamin levels in different healthy control groups based on age (<55 and ≥ 55 years), and no significant differences were found between these two groups (Supplementary Table 5). Further correlation analysis indicated that no significant association existed between serum vitamins (vitamin A, B1, B2, B9, B12, C, D, and E) and AAO, disease duration, and ALSFRS-score (Table 3).

**Table 3:** Correlation analysis between different vitamins and clinical characteristics of amyotrophic lateral sclerosis (ALS).

**Vitamins**	**AAO**		**Disease duration**		**ALSFRS-R score**	
	**^#^rs**	***p*-value**	**rs**	***p*-value**	**rs**	***p*-value**
Vitamin A	0.021	0.769	0.003	0.0.982	0.121	0.088
Vitamin B1	−0.017	0.814	−0.035	0.617	0.058	0.413
Vitamin B2	−0.118	0.093	−0.104	0.142	0.119	0.094
Vitamin B9	0.018	0.800	0.034	0.635	0.007	0.918
Vitamin B12	−0.052	0.458	−0.025	0.728	−0.059	0.408
Vitamin C	0.085	0.229	0.005	0.945	−0.067	0.348
Vitamin D	−0.046	0.512	−0.066	0.351	−0.011	0.879
Vitamin E	−0.056	0.426	0.103	0.145	−0.080	0.259

# * correlation coefficients*.

## Discussion

In the present study, lower levels of vitamin B2, B9, and C were found in patients with ALS and mimics compared to those in healthy controls. In addition, logistic regression results suggested that serum vitamin B2, B9, and C could exert protection against ALS. Thus, low levels of vitamin B2, B9, and C may be risk factors for ALS. Vitamin B2, also called riboflavin, is a precursor of the coenzymes flavin mononucleotide (FMN) and flavin adenine dinucleotide (FAD), which are essential in the mitochondrial electron transport chain and responsible for regenerating the antioxidant glutathione reductase. Thus, riboflavin acts indirectly as an antioxidant. Neurological abnormalities, such as ataxia and inability to stand, due to riboflavin deficiency have been reported (72). In addition, riboflavin transporter deficiency has been characterized as a cause of genetic recessive child-onset motor neuron disease with hearing loss, formerly described as Brown-Vialetto-Van-Lear syndrome (73). Thus, although serum vitamin B2 has not been researched in patients with ALS, the above evidence supports our results that low levels of vitamin B2 may be a risk factor for ALS.

Vitamin B9, also known as folic acid, can indirectly reduce the risk of multiple neurodegenerative diseases by reducing homocysteine levels in blood (74). Zhang et al. (75) found that folic acid treatment could significantly delay AAO and prolong the lifespan of transgenic ALS mice by attenuating plasma homocysteine levels. They also found that levels of tetrahydrofolate decreased significantly at the middle to late stages of the disease in a mouse model of ALS (27). Thus, low serum vitamin B9 may increase the risk of ALS by affecting homocysteine levels.

Vitamin C, as an important antioxidant, was reported to prolong the survival of familial ALS transgenic mice if vitamin C was administered before the onset of the disease (76). However, some studies found that neither supplement use nor high dietary intake of vitamin C was associated with a reduced risk for ALS (11, 19, 21). Moreover, Paraskevas et al. (77) reported that there was no significant difference in plasma and CSF vitamin C levels between patients with ALS and controls. However, their study only included a relatively small number of subjects (19 patients with ALS and 15 controls). In addition, the absorption of vitamin C could be altered by a variety of conditions, and there is a sigmoidal relationship between oral vitamin C dose and plasma vitamin C concentrations. Thus, the discrepancy between these studies and our study may be related to different models (animal model vs. patients with ALS), different forms of vitamin C (dietary intake vs. serum levels), and limited numbers of patients.

In the present study, serum vitamin A and E levels were significantly higher in patients with ALS compared to healthy controls. In addition, the ORs of vitamin A and E for ALS were significantly > 1, indicating that high levels of vitamin A and E were associated with an increased risk of ALS. However, pooled results from five cohort studies showed that a high intake of carotenoids (a main source of vitamin A) was associated with a reduced risk of ALS (11). In addition, Nieves et al. (78) reported that high intakes of antioxidants and carotenes from vegetables were associated with higher ALSFRS-R scores. Furthermore, several studies found that no significant relationship existed between β-carotene intake or vitamin A concentrations and the risk for ALS (17, 21, 23). Although most studies focused on the protective role of vitamin A as an antioxidant, the toxic effects regarding the redox environment and mitochondrial function have been reported (79). Furthermore, long-term dietary supplementation with retinoic acid has been reported to shorten the lifespan of an ALS mouse model (10). Thus, the conflicting results among these studies may be due to not only the limited number of patients and the heterogeneity of the patient cohorts but also that vitamin A itself has a complex function.

Similar to vitamin A, the results of vitamin E in our study were inconsistent with previous studies. Some past studies showed that serum vitamin E levels or high intake of vitamin E were unrelated to the risk of developing ALS (21, 22, 80). In addition, results from placebo-controlled double-blind studies revealed that the administration of vitamin E had no effect on slowing the disease progression of ALS (81, 82). Moreover, several studies found that regular use of a vitamin E supplement or higher serum vitamin E levels were associated with a low risk for ALS (17–20). However, the modest effect of serum vitamin E on reduced ALS risk was limited to subjects with low baseline serum vitamin E levels (17). In addition, vitamin E supplementation had no effect on the risk of ALS when the baseline serum vitamin E level was above the median. Therefore, the reason for the inconsistency may be attributed to the different baseline serum vitamin E levels, which seems to be neglected by many studies. The heterogeneity of the patient cohorts may also contribute to these discrepancies.

It is noteworthy that there were no differences in vitamin levels, except for vitamin E, between patients with ALS and mimics. This may be due to the similar pathogenesis, especially with regard to oxidative stress, between patients with ALS and mimics. It also indicates that vitamin level alterations are a common phenomenon among patients with neurodegenerative diseases, and is not specific to ALS. Although extensive studies have researched vitamins in different neurodegenerative diseases, few explored whether the alterations in vitamins were similar among neurodegenerative diseases. Our study may encourage more attention to be paid to this field. Interestingly, the two vitamins (A and E) that were found at higher levels in patients with ALS were fat-soluble, and the three vitamins (B2, B9, and C) that were found at lower levels in patients with ALS were water-soluble. These differences may be accounted for by issues in absorption and the metabolic pathways corresponding to the vitamins, but this needs to be confirmed and validated by further research. In addition, there were some interactions between vitamins. Vitamin A was inversely correlated with vitamin B1 and C, but positively correlated with vitamin B2; vitamin B1 and B9 were positively correlated with vitamin C; vitamin E was negatively correlated with vitamin C but positively correlated with vitamin B12 (Supplementary Table 2). Therefore, the complex interaction of vitamins and their synergism (30) should be taken into consideration when supplementing vitamins.

Noticeably, some patients with ALS and mimics suffered dysphagia, which may alter dietary intake, thus affecting the serological measurement of these vitamins. Although we did not find any significant differences in serum vitamins in patients with ALS who had onset with bulbar symptoms compared with those who had onset with other symptoms, we could not exclude the dietary effects on serum vitamins in the present study. In addition, we analyzed serum vitamins in patients without dysphagia, dysarthria, and sialorrhea and found that the alterations in vitamins show a similar trend to the alterations observed in the full sample analysis (data not shown). Therefore, we could at least conclude that dysphagia is not the reason for the differences in vitamin levels between patients with ALS and healthy controls in our study. In the present study, we found that vitamin C levels were lower in patients with early-onset compared to late-onset ALS; however, no significant correlation was found between each serum vitamin and AAO. Although we could not conclude the specific role of vitamin C in the AAO of ALS, our results suggest a possible association between low vitamin C levels and early-onset ALS. As low serum vitamin C might be a risk factor for ALS, supplementing vitamin C in patients with ALS may should be taken into consideration. Our study also observed no significant relationships between vitamin levels and onset sites, ALSFRS-R score, and disease duration. Similar to our findings, it has previously been reported that serum vitamin A and E levels in ALS were unrelated to the disease duration, severity, and even the form of onset (13, 23, 80). Therefore, although serum vitamins may be associated with the risk of ALS, further research regarding their role in ALS is still needed.

The strengths of this study included mimic enrollment and systematic evaluation of almost all vitamins at the same time in a large Chinese cohort of patients with ALS. Moreover, the analysis of associations between vitamins and disease conditions or sites of disease onset helped us to exclude their effects on vitamin levels. The contribution of vitamins C to early-onset ALS was also reported for the first time.

There were some limitations to our study. First, its retrospective nature could have caused selection bias. Second, this was a one-center survey-based study in which the subjects enrolled were limited to central and southern China. Third, some confounding factors, such as diet, were not included in this present study, which may affect our results about the alterations in serum vitamins. Moreover, despite the fact that our study provided information on the association between different vitamins and ALS, the role of some of these in ALS still requires further validation. Thus, multi-center prospective controlled studies are needed to evaluate the role of different vitamins in ALS.

In summary, for the first time, we have screened almost all vitamins in a large Chinese cohort of patients with ALS and explored the association between ALS and serum vitamins. In addition, we found that the trend of vitamin alterations in patients with ALS was similar to that in patients with other neurodegenerative diseases. Our study adds information to the literature on the role of vitamins in ALS and provides support for the clinical guidance on dietary and vitamin supplements in patients with ALS.

## Data Availability Statement

The datasets generated for this study are available on request to the corresponding author.

## Ethics Statement

The studies involving human participants were reviewed and approved by the Expert Committee of Xiangya Hospital, Central South University, China. Written informed consent to participate in this study was provided by the participants' legal guardian/next of kin.

## Author Contributions

MW analyzed data and wrote original draft. ZL, WS, YY, BJ, and XZ collected clinical data. LS, HJ, KX, and BT supervised the process. JW designed the study and edited the manuscript. All authors approved for the final version.

## Conflict of Interest

The authors declare that the research was conducted in the absence of any commercial or financial relationships that could be construed as a potential conflict of interest.
