# Supplementary material for: Association Between Vitamins and Amyotrophic Lateral Sclerosis: A Center-Based Survey in Mainland China
Source: Front Neurol. 2020 Jun 18;11:488. doi: 10.3389/fneur.2020.00488 (PMC7314934; doi:10.3389/fneur.2020.00488)
Supplement: Supplementary file 1 [file Data_Sheet_1.doc]

**Supplementary Table 1** Levels of different vitamins in patients with ALS, mimic controls, and healthy controls after control of age, sex, menopause, and serum cholesterol.

| Vitamins | ALS patients （N=193） | Mimics (N=149) | Healthy controls  (N=79) | p value |
| --- | --- | --- | --- | --- |
| Age (years) | 55.69±10.79 | 55.79±12.27 | 53.52±7.95 | 0.266 |
| Gender (Male/Female) | 131/62 | 82/67 | 52/30 | 0.051 |
| No. Menopause/total female | 48/62 | 45/60 | 26/30 | 0.439 |
| Cholesterol (mmol/L） | 4.74±0.97 | 4.53±0.93 | 4.78±0.75 | 0.994 |
| Vitamin A (umol/L) | 2.70 ± 0.85^*^ | 2.53±0.71 | 2.45±0.82^*^ | **0.025** |
| Vitamin B1 (nmol/L) | 121.25 ± 27.65 | 119.42±25.37 | 122.72±21.44 | 0.623 |
| Vitamin B2 (ug/L) | 7.81 ± 1.43^*^ | 7.42±1.10^#^ | 8.44±2.37^*#^ | **<0.0001** |
| Vitamin B9 (ug/L) | 6.56 ± 3.06^*^ | 7.72±5.10^#^ | 10.71±6.82^*#^ | **<0.0001** |
| Vitamin B12 (ug/L) | 0.52 ± 0.39 | 0.53±0.50 | 0.45±0.41 | 0.383 |
| Vitamin C (umol/L) | 31.69± 11.52^*^ | 33.90±10.06^#^ | 42.36±9.27^*#^ | **<0.0001** |
| Vitamin D (nmol/L) | 89.29± 63.24 | 87.66±27.67 | 94.87±81.90 | 0.663 |
| Vitamin E (ug/mL) | 7.54 ± 1.75^*$^ | 7.06±1.14^$^ | 6.84±1.34^*^ | **<0.0001** |

Values are shown as mean (±standard deviation). ^*^ denotes that a significant difference exists between patients with ALS and healthy controls; ^#^ denotes that a significant difference exists between mimic controls and healthy control; ^$^ denotes a significant difference existed between ALS patients and mimic. Multiple comparison were corrected with Bonferroni correction with p value less 0.017 considered to be significant.

**Supplementary table 2** Correlation analysis between different variables included in the logistic regression.

| Variable | Vitamin A | Vitamin B1 | Vitamin B2 | Vitamin B9 | Vitamin B12 | Vitamin C | Vitamin D | Vitamin E | Age | Gender | Cholesterol |
| --- | --- | --- | --- | --- | --- | --- | --- | --- | --- | --- | --- |
| Vitamin A | 1 |  |  |  |  |  |  |  |  |  |  |
| Vitamin B1 | -0.208* | 1 |  |  |  |  |  |  |  |  |  |
| Vitamin B2 | 0.159* | -0.015 | 1 |  |  |  |  |  |  |  |  |
| Vitamin B9 | -0.069 | 0.093 | 0.109 | 1 |  |  |  |  |  |  |  |
| Vitamin B12 | -0.090 | 0.079 | -0.010 | -0.044 | 1 |  |  |  |  |  |  |
| Vitamin C | -0.166* | 0.151* | -0.026 | 0.176* | -0.031 | 1 |  |  |  |  |  |
| Vitamin D | 0.107 | -0.017 | -0.003 | -0.063 | 0.068 | -0.067 | 1 |  |  |  |  |
| Vitamin E | 0.016 | 0.042 | 0.069 | -0.027 | 0.163* | -0.213* | -0.060 | 1 |  |  |  |
| Age | 0.040 | 0.043 | -0.058 | -0.081 | 0.009 | -0.024 | -0.064 | -0.003 | 1 |  |  |
| Gender | 0.027 | -0.006 | 0.012 | -0.066 | 0.016 | 0.002 | 0.071 | -0.028 | 0.070 | 1 |  |
| Cholesterol | -0.074 | -0.067 | 0.031 | 0.066 | -0.127* | -0.001 | -0.007 | -0.082 | 0.041 | -0.126* | 1 |

Note :the two-tailed p<0.05 level was accepted for significance testing. *denotes the correlation coefficient significant.

**Supplementary Table 3** Levels of different vitamins in ALS patients divided by age at onset after controlling of age, gender, and disease duration.

|  | Bulbar (N=26) | Upper limb (N=68) | Lower limb (N=73) | Multiple onset site (34) | *p* value |
| --- | --- | --- | --- | --- | --- |
| Age (years) | 60.39±9.89 | 55.45±10.17 | 55.32±11.60 | 53.25±10.15 | 0.08 |
| Gender (M/F) | 17/9 | 50/18 | 47/26 | 22/12 | 0.65 |
| Disease duration (months) | 15.54 ± 14.20 | 17.27 ± 17.44 | 17.80 ± 19.55 | 14.77 ± 13.83 | 0.82 |
| Vitamin A (umol/L) | 2.55±0.481 | 2.57±0.83 | 2.78±0.88 | 2.81±0.94 | 0.326 |
| Vitamin B1 (nmol/L) | 128.00±34.27 | 124.95±28.16 | 120.67±25.41 | 111.84±26.24 | 0.082 |
| Vitamin B2 (ug/L) | 7.48±1.10 | 7.79±1.65 | 7.77±1.40 | 8.11±1.95 | 0.373 |
| Vitamin B9 (ug/L) | 7.06±4.14 | 7.70±4.81 | 7.92±6.34 | 7.69±5.12 | 0.992 |
| Vitamin B12 (ug/L) | 0.56±0.41 | 0.53±0.40 | 0.47±0.37 | 0.68±0.51 | 0.097 |
| Vitamin C (umol/L) | 34.05±13.19 | 33.46±11.84 | 30.56±10.16 | 27.59±9.17 | 0.040 |
| Vitamin D (nmol/L) | 86.08±29.60 | 86.37±25.91 | 80.14±30.52 | 82.14±22.85 | 0.769 |
| Vitamin E (ug/mL) | 7.30±1.65 | 7.61±1.85 | 7.57±1.78 | 7.72±1.72 | 0.824 |

Values are shown as mean (±standard deviation).

**Supplementary Table 4** Levels of different vitamins in ALS patients divided by age at onset after controlling of gender and disease duration.

|  | Early onset ALS patients (N=105) | Late onset ALS patients (N=97) | *p* value |
| --- | --- | --- | --- |
| Gender (M/F) | 65/40 | 71/26 | 0.097 |
| Disease duration (months) | 17.85 ± 20.96 | 15.67 ± 11.99 | 0.361 |
| Vitamin A (umol/L) | 2.68±0.98 | 2.69±0.74 | 0.903 |
| Vitamin B1 (nmol/L) | 120.50±29.50 | 122.63±26.39 | 0.592 |
| Vitamin B2 (ug/L) | 7.95±1.54 | 7.64±1.24 | 0.122 |
| Vitamin B9 (ug/L) | 7.49±5.81 | 7.91±4.89 | 0.577 |
| Vitamin B12 (ug/L) | 0.55±0.44 | 0.52±0.39 | 0.659 |
| Vitamin C (umol/L) | 29.56±10.28 | 33.55±11.75 | **0.011*** |
| Vitamin D (nmol/L) | 84.35±29.31 | 83.78±25.65 | 0.884 |
| Vitamin E (ug/mL) | 7.70±1.84 | 7.44±1.68 | 0.303 |

Values are shown as mean (±standard deviation). * denotes p<0.05 were considered to be significant.

**Supplementary Table 5** Levels of different vitamins in health controls divided by age with the boundary of 55 years old adjusting for gender.

|  | Healthy controls age less than 55 years old (N=195) | Healthy controls age no less than 55 years old(N=35) | *p* value |
| --- | --- | --- | --- |
| Age (years) | 36.97±7.83 | 61.314±6.08 | - |
| Gender (M/F) | 102/93 | 20/15 | 0.558 |
| Vitamin A (umol/L) | 2.18±0.75 | 2.36±0.81 | 0.197 |
| Vitamin B1 (nmol/L) | 124.51±19.59 | 117.85±24.55 | 0.136 |
| Vitamin B2 (ug/L) | 8.01±1.91 | 8.38±2.41 | 0.319 |
| Vitamin B9 (ug/L) | 10.16±6.52 | 8.32±6.09 | 0.123 |
| Vitamin B12 (ug/L) | 0.47±0.42 | 0.46±0.41 | 0.875 |
| Vitamin C (umol/L) | 41.06±9.62 | 39.22±8.70 | 0.293 |
| Vitamin D (nmol/L) | 86.44±13.71 | 86.71±15.70 | 0.918 |
| Vitamin E (ug/mL) | 6.75±1.31 | 6.65±1.35 | 0.926 |

Values are shown as mean (±standard deviation).
